# Antigenic characterization and mapping of a conserved neutralizing epitope on VP1 of coxsackievirus A5

**DOI:** 10.3389/fimmu.2026.1837529

**Published:** 2026-05-13

**Authors:** Chen Wang, Wei-Ping Jin, Jie Wu, Jing Guo, Sha-Sha Qian, Xiao-Qi Chen, Shuo Shen

**Affiliations:** 1 Wuhan Institute of Biological Products Co., Ltd., Wuhan, China; 2 Hubei Provincial Vaccines Technology Innovation Center, Wuhan, China; 3 China Zhejiang Key Laboratory of Public Health Detection and Pathogenesis Research, Zhejiang Provincial Center for Disease Control and Prevention, Hangzhou, China

**Keywords:** coxsackievirus A5, epitope mapping, monoclonal antibody, passive protection, viral particles

## Abstract

**Background:**

Coxsackievirus A5 (CV-A5) has re-emerged as an important pathogen associated with hand, foot, and mouth disease (HFMD) in young children. However, the antigenic structure of CV-A5 and the key determinants responsible for neutralizing antibody responses remain largely undefined, hindering rational vaccine design and antibody-based intervention strategies.

**Methods:**

To investigate the relationship between viral particle composition and humoral immune responses, three forms of CV-A5 particles, including empty particles (EPs), full particles (FPs), A-particles (APs) were purified from the candidate vaccine strain CV-A5-3487, and artificial B-particles (BPs) were generated by heat treatment of FPs. These four particles were evaluated in mice. A mouse-adapted strain (CV-A5-611) was used to mimic natural infection for monoclonal antibody generation, and neutralizing hybridomas were rapidly screened using a fluorescent reporter virus (CV-A5-r611-iLOV). Linear epitope mapping was performed using truncated peptides and alanine scanning peptides, followed by sequence conservation analysis. Protective efficacy of the neutralizing antibody was evaluated in a lethal mouse challenge model.

**Results:**

Among the four particle types, FPs elicited the most potent neutralizing antibody responses, indicating that intact virions are critical for inducing effective humoral immunity. Hybridoma screening generated 617 ELISA-positive clones, of which 84 (13.61%) exhibited neutralizing activity against CV-A5. Ten neutralizing monoclonal antibodies (mAb) were obtained after subcloning, and only one antibody, 4D6, recognized a linear epitope. Epitope mapping identified the binding site within VP1 residues 155-169 (VPPGAPVPTGRDTFQ), with the minimal neutralizing core epitope defined as 158GAPVPTGR165. This epitope showed high sequence conservation (90.5%) among CV-A5 strains, suggesting a structurally constrained neutralization site. Passive transfer experiments demonstrated that mAb 4D6 conferred complete protection against lethal CV-A5 challenge at a dose of 20 μg per mouse, confirming its strong *in vivo* protective activity.

**Conclusion:**

Our study provides a systematic comparison of the immunogenicity of distinct CV-A5 particle forms and identifies a highly conserved linear neutralizing epitope on VP1 for the first time. The potent protective efficacy of mAb 4D6 highlights the importance of this epitope as a target for therapeutic antibody development and rational vaccine design, and provides new insights into the mechanisms of protective humoral immunity against CV-A5 infection.

## 1 Introduction

Hand, foot, and mouth disease (HFMD) is a common viral infectious disease that primarily affects infants and young children and poses a persistent public health burden in many regions. HFMD is caused by a variety of enteroviruses belonging to the genus *Enterovirus* within the family *Picornaviridae*, among which enterovirus A71 (EV-A71) and coxsackievirus A16 (CV-A16) have historically been regarded as the major etiological agents (1). However, following the widespread introduction of the inactivated EV-A71 vaccine in 2015, the proportion of HFMD cases attributable to EV-A71 in China, including mild, severe, and fatal cases, decreased markedly from 62.8% to 32.8% (2). Concurrently, the epidemiological profile of HFMD has undergone a noticeable shift, with an increasing proportion of cases associated with non-EV-A71 enteroviruses (3, 4). Among these emerging pathogens, coxsackievirus A5 (CV-A5) has been implicated in outbreaks accompanied by neurological complications and severe clinical manifestations, underscoring the need for a deeper understanding of its virological and immunological characteristics. CV-A5 belongs to the genus *Enterovirus* within the family *Picornaviridae* and is a non-enveloped virus with a positive-sense, single-stranded RNA genome of approximately 7.4 kb. The viral capsid exhibits icosahedral symmetry and is composed of 60 protomers, each containing four structural proteins (VP1-VP4) (5). During the viral life cycle, enteroviruses particles undergo a series of dynamic structural transitions during viral assembly, maturation, and uncoating. These processes give rise to multiple distinct particle forms, including empty particles (EPs), mature full particles (FPs), and uncoating intermediates such as A-particles and B-particles (6–8). Previous studies on poliovirus, EV-A71, and other picornaviruses have demonstrated that these distinct particle forms differ in antigenic structure and immunogenicity. In general, mature FPs preserve the native capsid conformation and induce stronger neutralizing antibody responses than EPs (9), whereas structurally altered particles may expose cryptic or non-protective epitopes. Despite these observations, the composition, heterogeneity, and immunological relevance of different CV-A5 particle populations remain insufficiently investigated, although the immunogenicity of EPs and FPs of the CV-A5-vN20 strain have been preliminarily characterized in our previous study (10).

Protective immunity against enteroviruses is primarily mediated by neutralizing antibodies targeting antigenic epitopes on the viral capsid, especially the surface-exposed protein VP1. Viral antigenic epitopes are generally classified as either linear or conformational epitopes. Linear epitopes consist of continuous amino acid sequences, whereas conformational epitopes are formed by amino acid residues that are discontinuous in primary sequence but spatially adjacent in the three-dimensional structure of the protein (11). Extensive epitope mapping has been performed for EV-A71, CV-A16, and CV-A10 (12–16); however, the antigenic landscape of CV-A5 remains largely undefined, and no neutralizing epitope has been clearly characterized to date. This lack of knowledge limits the rational design of vaccines and antibody-based therapeutics targeting this emerging HFMD-associated virus.

The efficient generation and identification of neutralizing monoclonal antibodies against enteroviruses remains technically challenging. Conventional immunization strategies often rely on inactivated viruses, which may not fully preserve native capsid conformation and frequently result in a high proportion of non-neutralizing antibodies. Moreover, traditional neutralization assays based on cytopathic effects are labor-intensive and time-consuming, limiting the throughput of antibody screening. Therefore, optimized immunization approaches that better mimic natural infection, together with rapid and functional screening platforms, are therefore required to improve the efficiency of neutralizing antibody discovery.

In the present study, we systematically characterized the structural heterogeneity and immunogenicity of different CV-A5 particle populations and evaluated their contributions to neutralizing antibody induction. By combining live virus priming with boosting using purified full particles and employing a fluorescent reporter virus-based neutralization assay, we established an efficient platform for the generation and screening of CV-A5-neutralizing monoclonal antibodies. Using this approach, we identified a linear epitope-specific neutralizing antibody, mAb 4D6, and mapped its binding site to a previously unreported conserved region within VP1. Importantly, we further demonstrated that this antibody conferred dose-dependent post-exposure protection in a lethal CV-A5 mouse model. Together, these findings provide new insights into CV-A5 antigenicity and neutralization mechanisms and offer valuable guidance for CV-A5-specific vaccine and antibody-based therapeutic development.

## 2 Materials and methods

### 2.1 Mice, cells, and viruses

Kunming (KM) mice (14 days old) and BALB/c mice (14 days old and 6–8 weeks old) of specific pathogen-free (SPF) grade were used in this study.

African green monkey kidney cells (Vero) and human rhabdomyosarcoma cells (RD) were obtained from the American Type Culture Collection (ATCC) and cultured as previously described (10). Mouse myeloma SP2/0 cells were provided by the Antibody Research Laboratory of WIBP.

The CV-A5 candidate vaccine strain CV-A5–3487 was used in this study (10), and its complete genome sequence has been deposited in GenBank (accession number MN663160). The mouse-adapted lethal challenge strain CV-A5–611 was obtained by plaque purification of CV-A5-M14 (10) (GenBank accession number MW079817). The fluorescent reporter virus CV-A5-r611-iLOV was constructed and preserved in our laboratory as described previously (17). Viral titers were determined as 50% cell culture infective doses (CCID_50_) using the Reed-Muench method (18).

### 2.2 Purification of CV-A5 particles

Virus-containing cell culture supernatants were subjected to three freeze-thaw cycles and clarified by centrifugation at 10,000 × g for 1 h at 4 °C to remove cellular debris. The supernatants were subsequently filtered and concentrated to a final volume of 180 mL using a 100-kDa tangential flow filtration system (Sartorius).

Virus purification was performed by ultracentrifugation through a 20% (w/v) sucrose cushion at 144,000 × g for 3 h at 4 °C using a Beckman SW28 rotor. The resulting pellets were resuspended in phosphate-buffered saline (PBS, pH 7.2) and incubated overnight at 4 °C for complete dissolution. The virus suspensions were further purified by cesium chloride (CsCl) density gradient ultracentrifugation at an initial density of 1.31 g/mL using a Beckman SW41 rotor at 155,000 × g for 22 h at 4 °C. After centrifugation, visible opalescent bands corresponding to EPs, FPs, and APs were collected using a syringe and pelleted by centrifugation at 260,000 × g for 3 h at 4 °C to remove CsCl. The pellets were resuspended in PBS and stored at -70 °C until use.

### 2.3 Characterization of CV-A5 particles

Purified CV-A5 particles (EPs, FPs, and APs) were analyzed by sodium dodecyl sulfate-polyacrylamide gel electrophoresis (SDS-PAGE), Western blotting (WB), and negative-staining transmission electron microscopy (TEM). Total protein concentrations of individual fractions were determined using a Pierce bicinchoninic acid (BCA) protein assay kit (Micro BCA, Thermo Fisher Scientific). The purity of each particle preparation was calculated based on Coomassie Brilliant Blue-stained SDS-PAGE gels and WB results using ImageJ software.

Rabbit polyclonal antibodies against CV-A5 whole virions, VP2, VP3, and VP4 were prepared in our laboratory and used at a dilution of 1:7,000. Viral particles were diluted to 100-200 μg/mL in PBS (pH 7.2). Carbon-coated copper grids were incubated with 20 μL of purified viral particles for 5 min, negatively stained with 1% phosphotungstic acid (pH 7.0) for 5 min, air-dried overnight, and examined using a transmission electron microscope (Hitachi).

To evaluate humoral immunogenicity, six- to eight-week-old female BALB/c mice were randomly divided into six groups (n = 8 per group) and immunized intraperitoneally (i.p.) on days 0 and 14. Five groups were immunized with purified EP-1, EP-2, FP, AP, or BP at a dose of 0.1 μg per mouse in a volume of 500 μL, respectively. The control group received aluminum hydroxide [Al(OH)_3_] at the same volume. Serum samples were collected on day 28, heat-inactivated at 56 °C for 30 min, and stored at -20 °C until analysis. Neutralizing antibody (NtAb) titers were determined as previously described (10).

### 2.4 Generation of hybridoma cells

To generate monoclonal antibodies (mAbs) with neutralizing activity, BALB/c mice (14 days old) were infected with the mouse-adapted strain CV-A5–611 to mimic natural infection. Mice were inoculated either intraperitoneally or by gavage at doses of 1 × 10^6^ CCID_50_ or 1 × 10^7^ CCID_50_ per mouse, respectively. Booster immunizations were administered at 28 days of age using the same routes at doses of 1 × 10^8^ CCID_50_ or 1 × 10^9^ CCID_50_ per mouse, respectively.

Twenty-one days later, mice were further boosted intraperitoneally with purified FPs at a dose of 50 μg per mouse. Serum samples were collected from the orbital sinus seven days after the third immunization to assess antibody titers. Mice exhibiting high ELISA and neutralizing antibody titers were selected for splenic booster immunization three days prior to cell fusion, using 10 μg of purified FPs per mouse.

Splenocytes were fused with SP2/0 myeloma cells using standard polyethylene glycol-mediated fusion. Hybridoma cells were cultured in RPMI 1640 medium supplemented with 20% fetal bovine serum (FBS) and hypoxanthine-aminopterin-thymidine (HAT) for 8 days, followed by culture in RPMI 1640 containing 20% FBS and hypoxanthine-thymidine (HT) for an additional 2–4 days.

### 2.5 Indirect ELISA screening for mAbs

To assess the specificity with CV-A5, purified CV-A5 FPs were diluted to 1 μg/mL in PBS (pH 7.2), and 100 μL of antigen was added to each well of 96-well microtiter plates. Plates were coated overnight at 4 °C in 50 mM carbonate buffer (pH 9.6). After washing three times with PBST (PBS containing 0.05% Tween 20), plates were blocked with PBST containing 1% bovine serum albumin (BSA) and 20% sucrose at 37 °C for 1 h. Hybridoma culture supernatants were diluted in blocking buffer and added to the plates in triplicate (100 μL per well). Serum collected prior to cell fusion served as the positive control, whereas serum collected before immunization served as the negative control. Plates were incubated at 37 °C for 1 h and washed five times with PBST. Horseradish peroxidase (HRP)-conjugated secondary antibodies (a mixture of goat anti-mouse IgG, IgM, and IgA) were diluted 1:10,000, added to the plates, and incubated at 37 °C for 1 h. Substrate solutions A and B (Wantai Biopharm) were added in equal volumes (50 μL per well) and incubated at 37 °C for 30 min. Reactions were terminated by the addition of 50 μL stop solution, and absorbance was measured at 450 nm using a microplate reader (Thermo Fisher Scientific).

### 2.6 Neutralization assay of hybridoma cell supernatants

Neutralizing activity of hybridoma supernatants was assessed using the fluorescent reporter virus CV-A5-r611-iLOV (17). Hybridoma supernatants that tested positive by ELISA were diluted 1:8 and 1:64, and 50 μL of each dilution was added to 96-well plates in duplicate. An equal volume of minimum essential medium (MEM) was added to cell control wells. Virus was diluted to 100 CCID_50_/50 μL and added to each well. After incubation at 37 °C for 2 h, RD cells (1 × 10^4^ cells in 100 μL) were added to each well. Virus back-titration was performed to ensure that viral input ranged from 32 to 320 CCID_50_/50 μL. After 36 h of incubation, neutralizing activity was determined by fluorescence microscopy. Wells lacking detectable fluorescence at a dilution of 1:8 were considered positive for neutralizing activity.

### 2.7 Western blotting assay of hybridoma cell supernatants

To assess specificity and reactivity of the mAbs secreted by hybridoma cell supernatants, purified CV-A5 FPs (1 ng per lane) were used in Western blotting. Hybridoma supernatants that tested positive by ELISA were diluted at 1:10 and used as primary antibodies, and a mixture of HRP-conjugated goat anti-mouse IgG, IgM, and IgA (1:10,000) was used as the secondary antibody.

### 2.8 Characterization of ten monoclonal antibodies

Antibody isotypes were identified by ELISA using eight different HRP-conjugated secondary antibodies. The subclass corresponding to the highest absorbance value was assigned as the antibody isotype. To assess the cross-reactivity of ten mAbs, the ELISA plates were coated with purified CV-A2, CV-A4, CV-A5, CV-A6, CV-A10, CV-A16, EV-A71, echovirus 11 (Echo11) or poliovirus particles at a concentration of 1 μg/mL (100 μL per well). Specificity and reactivity of the mAbs were further confirmed by Western blotting using purified CV-A5 FPs (1 ng per lane). Purified mAbs (50 μg/mL) were used as primary antibodies, and a mixture of HRP-conjugated goat anti-mouse IgG, IgM, and IgA (1:10,000) was used as the secondary antibody.

### 2.9 Epitope mapping of mAb 4D6 using synthetic peptides

The specificity of mAb 4D6 for CV-A5 VP1 was initially confirmed by ELISA and Western blotting using purified inactivated antigens from CV-A2, CV-A4, CV-A5, CV-A6, CV-A10, CV-A16, EV-A71, echovirus 11, and poliovirus.

To identify the precise binding epitope of mAb 4D6, a total of 39 synthetic peptides (14–18 amino acids in length) spanning the entire VP1 protein of CV-A5 were synthesized (GenScript). Adjacent peptides overlapped by 10 amino acids. These peptides were screened by indirect ELISA to identify the epitope-containing region. Based on the initial results, a series of truncated peptides and alanine-substituted peptides covering the identified region were synthesized to define the minimal binding epitope and critical residues required for mAb 4D6 binding.

### 2.10 Sequence alignment of the mAb 4D6 epitope

To assess conservation of the identified linear epitope among CV-A5 strains, the amino acid sequence of the mAb 4D6 epitope was aligned with CV-A5 sequences retrieved from the NCBI enterovirus protein database (taxid: 12059) using SnapGene software.

### 2.11 Passive protection assay of mAb 4D6 *in vivo*

The titer of the mouse-adapted strain CV-A5–611 was determined in RD cells prior to challenge experiments. For determination of the median lethal dose (LD_50_), 14-day-old Kunming mice were randomly divided into five groups (n = 10 per group) and inoculated intraperitoneally with 10-fold serial dilutions of CV-A5-611 (3.16 × 10^7^, 3.16 × 10^6^, 3.16 × 10^5^, or 3.16 × 10^4^ CCID_50_ per mouse in 300 μL). Control mice received an equal volume of MEM. Survival was monitored daily for 14 days post-infection, and the LD_50_ was calculated using the Reed-Muench method (18).

For passive protection experiments, 14-day-old Kunming mice were divided into five groups (n = 10 per group) and challenged intraperitoneally with CV-A5–611 at a dose of 3.16 × 10^7^ CCID_50_ per mouse. Two hours post-challenge, mice received intraperitoneal injections of purified mAb 4D6 at doses of 60, 20, 6.67, or 2.22 μg in 100 μL PBS, respectively. Control mice received PBS alone. Clinical symptoms and survival were monitored daily for 14 consecutive days.

### 2.12 Statistical analysis

Neutralizing antibody titers, ELISA data, survival rates, antibody isotypes, EC_50_ values, and LD_50_ values were analyzed using GraphPad Prism 8 software (GraphPad Software Inc., USA). Statistical significance was assessed by paired t-test. Statistical significance was indicated as follows: ns, not significant (*P* ≥ 0.05); *, 0.01 ≤ *P* < 0.05; **, *P* < 0.01; ***, *P* < 0.001 and ****, *P* < 0.0001.

## 3 Results

### 3.1 Purification and characterization of distinct CV-A5 particle forms

**Figure 1:**
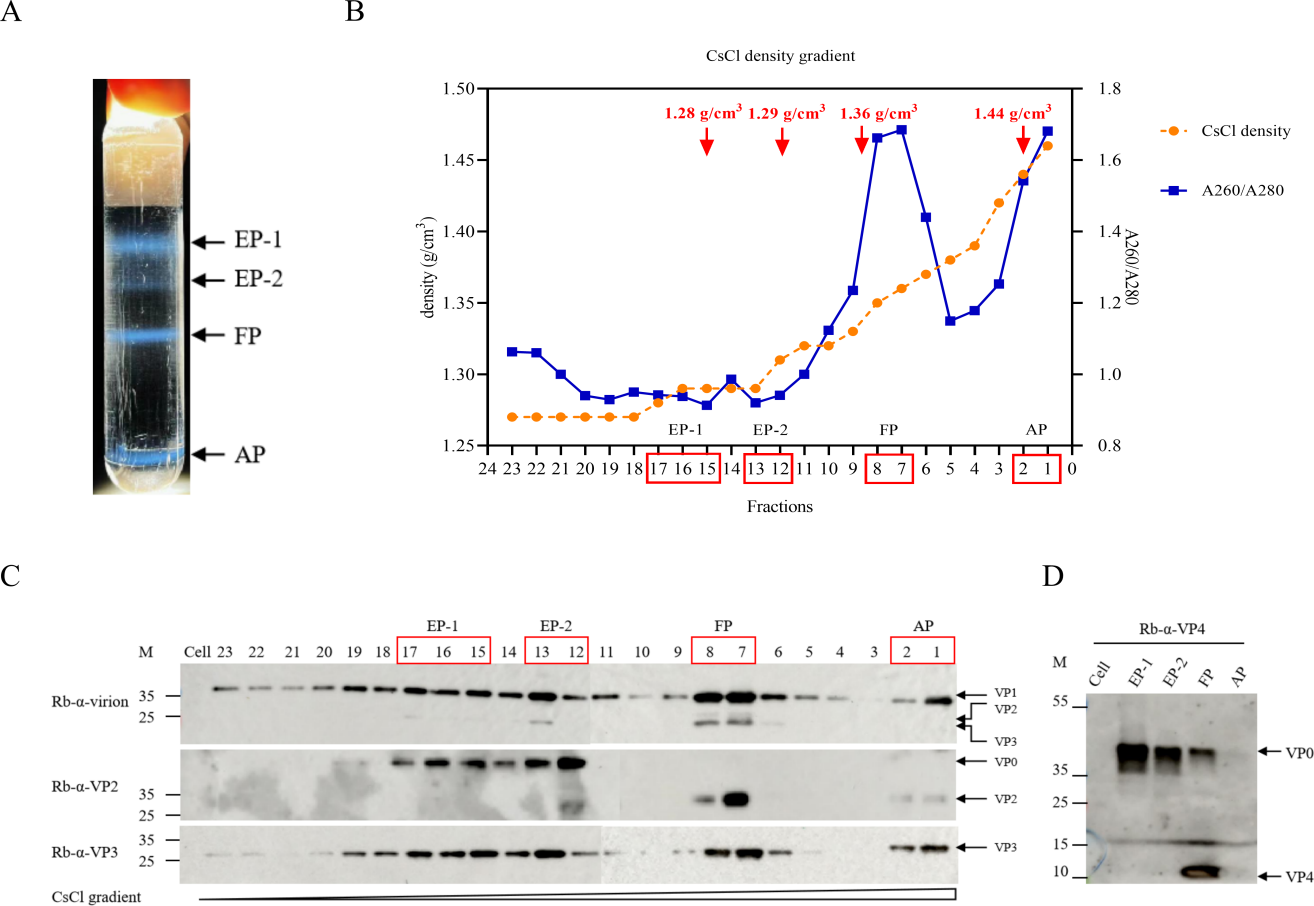
Purification and characterization of distinct CV-A5 particle populations. CV-A5 particles were purified by CsCl gradient ultracentrifugation. A total of 23 fractions were collected from the CsCl gradient. **(A)** The positions of empty particles (EP-1, EP-2), full particles (FP), and A-particles (AP) are indicated. **(B)** Absorbance ratios at 260 nm/280 nm (blue line) and CsCl density values (orange line) of the 23 fractions. Red arrows indicate the CsCl densities corresponding to the four particle types. Fractions within the red box were pooled for further analysis. **(C)** Western blotting analysis of the 23 fractions and mock-infected Vero cells, using antibodies against CV-A5 virion, VP2, and VP3. The molecular weight markers (M) are shown on the left, and the detected proteins are indicated on the right. **(D)** Western blotting analysis of the four pooled fractions and mock-infected Vero cells, using an anti-VP4 antibody.

To investigate the structural heterogeneity of CV-A5 particle, cell culture lysate of the CV-A5–3487 strain, an RD cell-isolated and Vero-adapted clinical isolate obtained from rectal swabs of an HFMD patient in Xiangyang, China in 2017 (10), was subjected to cesium chloride (CsCl) density gradient ultracentrifugation. Four distinct opalescent bands were observed in the gradient and were provisionally designated as EP-1, EP-2, FP, and AP, respectively (**Figure 1A**).

To determine the physicochemical properties of these bands, 23 fractions (500 μL per fraction) were sequentially collected sequentially from the bottom of the centrifuge tube and analyzed. Measurement of CsCl buoyant densities revealed values of 1.28 g/cm³, 1.29 g/cm³, 1.36 g/cm³, and 1.44 g/cm³, from top to bottom, corresponding to the four visible bands (**Figure 1B**). Analysis of nucleic acid content based on OD_260_/OD_280_ ratios demonstrated prominent peaks in fractions 1-2 (1.681) and 7-8 (1.662), indicating the presence of viral RNA and identifying these fractions as A-particles (APs) and full particles (FPs), respectively. In contrast, fractions 12–13 and 15–17 exhibited markedly lower OD_260_/OD_280_ ratios (0.941 and 0.913, respectively), consistent with the absence of viral genomic RNA. Based on their distinct buoyant densities and lack of nucleic acids, particles in fractions 12–13 and 15–17 were designated as two structurally distinct empty particle populations, termed EP-2 and EP-1, respectively.

Western blotting analysis further confirmed the structural composition of these particles. Fractions corresponding to EP-1 and EP-2 contained VP0, VP1, and VP3, demonstrating the presence of uncleaved VP0 and confirming their identity as immature empty particles (**Figure 1C**). In contrast, fractions corresponding to FPs and APs contained VP1, VP2, and VP3, indicating that VP0 cleavage had occurred in these particles. Notably, VP1 and VP3 detected in fractions 18–23 at the top of the gradient were attributed to dissociated protomers generated during particle depolymerization (**Figure 1C**). Additional probing with anti-VP4 antibodies revealed that EP-1 and EP-2 lacked VP4, whereas FPs contained abundant VP4, consistent with their identity as mature infectious virions. In contrast, APs showed loss of VP4, supporting their classification as uncoating intermediates formed following VP4 externalization (**Figure 1D**).

**Figure 2:**
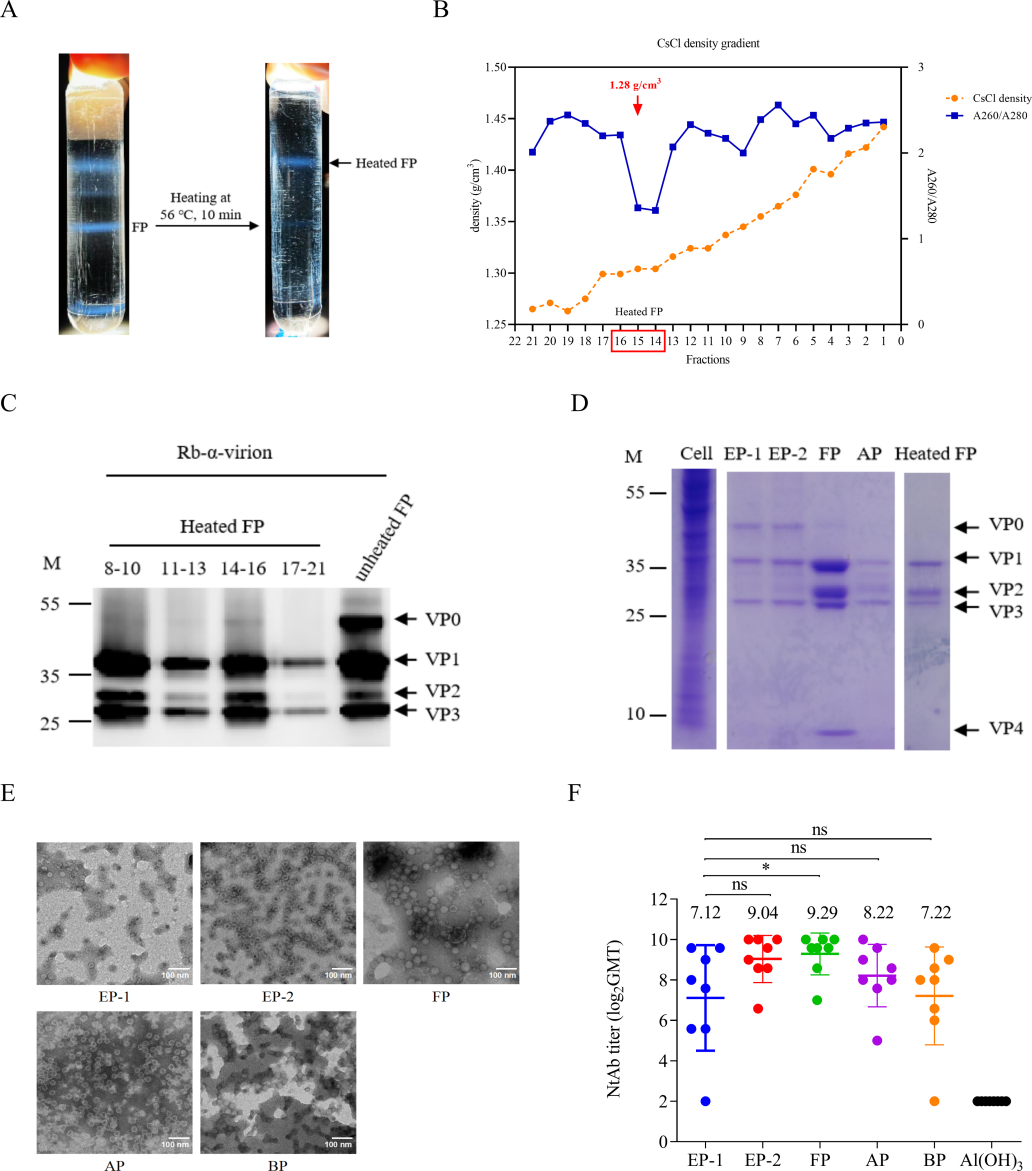
Purification and characterization of the heat-treated full particles (FPs) and other particles. CV-A5 FPs were heat-treated at 56 °C for 10 minutes, followed by RNase treatment and CsCl gradient ultracentrifugation. A total of 21 fractions were collected. **(A)** The positions of unheated and heat-treated full particles are indicated. **(B)** Absorbance ratios at 260 nm/280 nm (blue line) and CsCl density values (orange line) of the 21 fractions of heated FPs. Red arrows indicate the CsCl density corresponding to the heated FPs. Fractions within the red box were pooled and named heated FPs (BPs). **(C)** Western blotting analysis of unheated and heat-treated FPs, using an anti-CV-A5 virion antibody. **(D)** SDS-PAGE (4-20%) analysis of proteins from mock-infected Vero cells, EP-1, EP-2, FP, AP and heated FPs, stained with Coomassie brilliant blue. The detected proteins (VP0, VP1, VP2, VP3, and VP4) are indicated. **(E)** Transmission electron microscopy (TEM) images of the purified EP-1, EP-2, FP, AP and heated FPs (BP). Scale bar = 100 nm. **(F)** Humoral immune responses of the five purified particles. Six- to eight-week-old BALB/c mice were inoculated intraperitoneally (i.p.) on day 0 and 14 with EP-1, EP-2, FP, AP and BP at a dose of 0.1 μg per injection. Mice injected with Al(OH)_3_ alone served as the negative control. Bleeding was performed on day 28. Neutralizing antibody (NtAb) titers were presented as the geometric mean titer (GMT) ± the standard error of the mean (SEM) from eight mice per group. Each symbol represented a mouse, and the NtAb titers below 8 were assigned to 2 for the convenience of presentation. The GMT of each group was calculated and represented by the numbers above the symbols. A paired t-test was used to compare the significant differences between the EP-1 group and each of the other groups: ns, not significant (*P* ≥ 0.05); *0.01 ≤ *P* < 0.05.

To investigate the effect of thermal treatment on CV-A5 full particles, purified FPs were heated at 56 °C for 10 min, followed by RNase treatment and CsCl gradient centrifugation. This procedure resulted in the formation of a single opalescent band (**Figure 2A**), with a markedly reduced buoyant density of 1.28 g/cm³ compared with untreated FPs (1.36 g/cm³) (**Figure 2B**). The disappearance of the OD_260_/OD_280_ peak confirmed the loss of viral RNA from these particles. Western blotting analysis further showed a reduced relative amount of VP0 in heat-treated particles compared with untreated FPs, indicating that elevated temperature induced genome release and conformational rearrangements associated with particle destabilization (**Figure 2C**). These particles were therefore defined as artificially generated B-particles (BPs).

After removal of CsCl, particle purity was assessed by SDS-PAGE. EP-1 and EP-2 exhibited three major protein bands corresponding to VP0, VP1, and VP3; FP displayed four bands corresponding to VP1, VP2, VP3, and VP4; A faint VP0 band was also observed in the FP lane, which was consistent with previous reports indicating that a small proportion of VP0 may remain uncleaved in mature picornavirus particles (10); AP contained VP1, VP2, and VP3; and BP (Heated FP) lacked VP4 and viral RNA (**Figure 2D**). Densitometric analysis demonstrated high purity of the preparations, with purities of 85.5% (EP-1), 85.6% (EP-2), 98.3% (FP), 90.3% (AP), and 95.7% (BP).

Transmission electron microscopy further confirmed the structural differences among the five particle types. Compared with the compact morphology of FPs, EP-1, EP-2, AP, and BP exhibited expanded and less compact structures with increased electron density, consistent with capsid relaxation and increased permeability of CsCl (**Figure 2E**). A small proportion of EP-like particles was observed within FP preparations, which was very similar to those of poliovirus studies in which a trace amount of VP0 was found in the FP particles (19, 20). This result is likely due to the simultaneous generation of EPs and FPs during the cell culture, for which complete isolation of these two particles is difficult to achieve during downstream purification (20).

To compare the immunogenicity of the different particle types, BALB/c mice were immunized intraperitoneally with EP-1, EP-2, FP, AP, or BP. All particles elicited detectable neutralizing antibody responses (**Figure 2F**). The relative immunogenicity ranking as EP-1 < BP < AP < EP-2 < FP. Full particles induced the strongest neutralizing antibody responses, whereas EP-1 exhibited the weakest immunogenicity, indicating that both genome encapsidation and preservation of native capsid conformation critically contribute to optimal antigen presentation and induction of protective humoral immunity.

### 3.2 Generation of monoclonal antibodies in mice

To enhance humoral immune responses and increase the likelihood of obtaining monoclonal antibodies with neutralizing activity, an immunization strategy involving priming with live virus followed by boosting with purified viral particles was employed. The mouse-adapted CV-A5–611 strain was selected for immunization via intraperitoneal injection or oral gavage to simulate natural infection in BALB/c mice. Full particles were used for the second booster immunization and the final intrasplenic injection, as they exhibited higher immunogenicity than EPs in the present study, consistent with previous reports on CV-A2, CV-A4, and CV-A5 (10, 21, 22).

Following primary immunization, mice in the gavage group developed mild clinical signs, including piloerection, at 3 days post-infection (dpi), which gradually resolved without mortality. In contrast, mice immunized via intraperitoneal injection exhibited earlier and more pronounced symptoms, including piloerection and hindlimb weakness as early as 2 dpi, with one mouse succumbing within 7 days post-infection. The remaining mice recovered, confirming successful viral infection and immune priming via both routes.

Serological analysis after completion of the third immunization regimen revealed marked differences in antibody responses between the two groups. In the gavage group, the mean ELISA binding antibody titer was approximately 10³, with a mean neutralizing antibody titer of 64 (2^6^). In contrast, mice immunized intraperitoneally exhibited substantially higher humoral responses, with a mean ELISA binding titer exceeding 10^5^ and a mean neutralizing antibody titer of 384 (2^8^·^5^) (data not shown). These results indicate that the route of immunization strongly influences the magnitude of humoral immune responses. Based on these findings, a mouse from the intraperitoneal immunization group displaying the highest neutralizing antibody titer was selected for splenic booster immunization and subsequent hybridoma fusion. This optimized strategy combining live virus priming with boosting using purified full particles provided a strong humoral response and created favorable conditions for efficient generation of neutralizing monoclonal antibodies.

### 3.3 Screening and characterization of neutralizing monoclonal antibodies

Hybridoma culture supernatants were initially screened by indirect ELISA using purified CV-A5 full particles (FPs) as the coating antigen. From a total of 1,860 hybridoma clones obtained after fusion, 617 clones (33.17%) exhibited positive ELISA reactivity against CV-A5 FPs, indicating successful induction of CV-A5-specific antibody responses (data not shown). These ELISA-positive clones were subsequently subjected to functional screening for neutralizing activity using the fluorescent reporter virus CV-A5-r611-iLOV.

**Figure 3:**
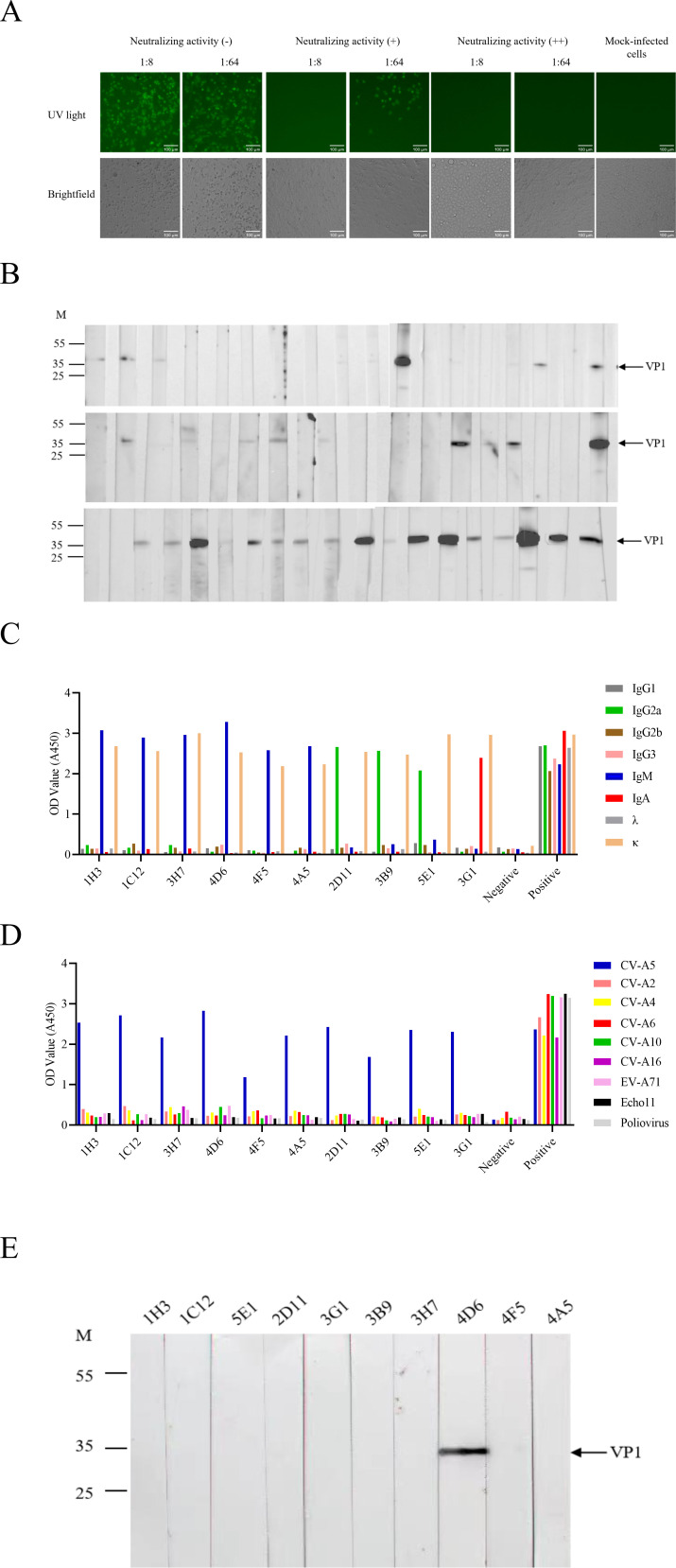
Screening and identification of neutralizing monoclonal antibodies (mAbs) against CV-A5. **(A)** Representative neutralization assays using CV-A5-r611-iLOV to identify neutralizing mAbs. Hybridoma cell supernatants were diluted 1:8 or 1:64 and inoculated with CV-A5-r611-iLOV for 36 hours. Autofluorescence was observed; wells without fluorescence at 1:8 dilution were considered neutralizing-positive (+), and wells with fluorescence were considered negative (−). Scale bar = 100 μm. **(B)** Representative Western blotting analysis of hybridoma supernatants to identify proteins recognized by the mAbs. Cell supernatants were diluted 1:10 and used as primary antibodies, with goat anti-mouse IgA, IgM, and IgG (1:10,000) as the secondary antibody. VP1 is indicated by an arrow. **(C)** Isotype determination of ten neutralizing mAbs. **(D)** ELISA analysis to assess the specificity of ten neutralizing mAbs against CV-A5. Plates were coated with purified particles from 10 enterovirus serotypes, with pre-immune mouse serum and rabbit antiserum used as negative and positive control, respectively. **(E)** Western blotting analysis of ten neutralizing mAbs. Protein of purified CV-A5 FPs (1 ng per lane) were separated by SDS-PAGE (4-20%) and probed with 50 μg/mL of 10 purified mAbs. Molecular weight markers (M) in kDa and viral VP1 protein are indicated.

Using this rapid fluorescence-based neutralization assay, 84 hybridoma clones (13.61% of ELISA-positive clones) were identified as secreting neutralizing antibodies within 24–36 h, as evidenced by complete inhibition of green fluorescence in infected RD cells at 1:8 dilution (**Figure 3A**). This screening strategy markedly accelerated identification of functional neutralizing antibodies compared with conventional cytopathic effect-based assays.

To distinguish antibodies recognizing linear versus conformational epitopes, all 617 ELISA-positive hybridoma supernatants were further analyzed by Western blotting under denaturing conditions. As shown in **Figure 3B**, 189 antibodies (30.63%) recognized VP1, indicating binding to linear epitopes, whereas the remaining 428 antibodies (69.37%) failed to detect viral proteins, consistent with recognition of conformational epitopes.

Based on combined ELISA, neutralization, and Western blotting results, hybridoma clones of interest were subjected to two rounds of limiting dilution subcloning. Ultimately, ten stable hybridoma cell lines secreting CV-A5-specific neutralizing monoclonal antibodies were obtained and designated 1H3, 1C12, 2D11, 3B9, 3H7, 3G1, 4A5, 4D6, 4F5, and 5E1.

Isotype analysis revealed that the ten monoclonal antibodies belonged to three immunoglobulin subclasses, IgA, IgG2a, and IgM, and all possessed kappa light chains (**Figure 3C**). Indirect ELISA demonstrated that all ten monoclonal antibodies bound CV-A5 FPs with high affinity, although binding titers varied among individual antibodies (**Figure 3D**). Importantly, none of the antibodies exhibited cross-reactivity with echovirus 11 or poliovirus, indicating high specificity for CV-A5 (**Figure 3D**).

Western blotting analysis further demonstrated that only one monoclonal antibody, 4D6, recognized VP1 under denaturing conditions (**Figure 3E**), indicating binding to a linear epitope. In contrast, the remaining nine neutralizing antibodies did not detect VP1 or other capsid proteins by Western blotting, suggesting that they recognize conformational epitopes. Among the ten neutralizing monoclonal antibodies, 4D6 was the sole antibody exhibiting both linear epitope recognition and potent neutralizing activity, and was therefore selected for detailed epitope mapping and functional characterization. A summary of the screening outcomes, including ELISA positivity, neutralizing activity, Western blotting reactivity, and epitope classification, is provided in **Table 1**.

**Table 1:** Summary of antigen-binding properties and neutralizing activities of CV-A5-specific antibodies secreted by hybridoma cells.

Antibody characteristic*	No. of cell lines	Proportion (%)
Epitope recognition		
Linear VP1 epitope	189	30.63
Conformational epitope	428	69.37
Functional properties		
Neutralization-positive	84	13.61

*Antibody reactivity was initially determined by indirect ELISA using purified CV-A5 full particles (FPs). Neutralizing activity was assessed using a fluorescence-based neutralization assay. Linear epitope recognition was defined by positive detection of VP1 under denaturing conditions by Western blotting, whereas antibodies lacking Western blotting reactivity were classified as conformational epitope-specific.

### 3.4 Characterization of mAb 4D6 by indirect ELISA, WB, and *in vitro* neutralizing assays

**Figure 4:**
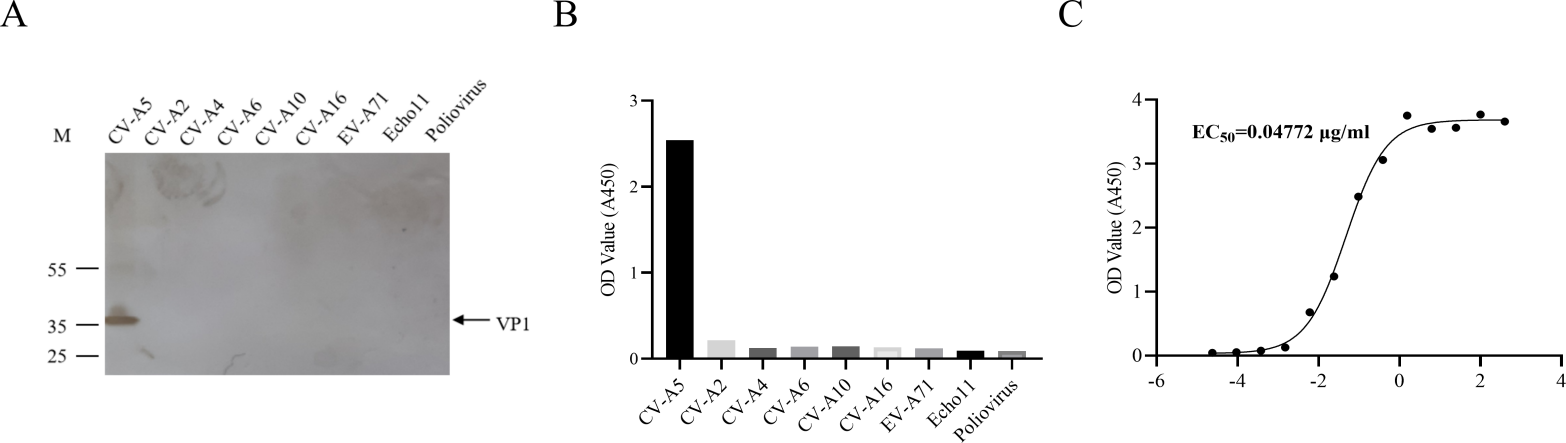
Specificity and binding activity of monoclonal antibody (mAb) 4D6. **(A)** Western blotting analysis to identify the specificity of mAb 4D6. Purified particles from nine enterovirus serotypes were separated by SDS-PAGE (4-20%), and mAb 4D6 (50 μg/mL) was used as the primary antibody. Goat anti-mouse IgM was used as the secondary antibody. VP1 is indicated by an arrow. **(B)** ELISA analysis to assess the binding activity of mAb 4D6. Plates were coated with purified particles from 9 enterovirus serotypes, and mAb 4D6 (50 μg/mL) was used to detect binding. **(C)** ELISA analysis of mAb 4D6’s binding activity to CV-A5 VP1.

To determine whether mAb 4D6 recognizes a linear or conformational epitope, Western blotting analysis was performed using purified CV-A5 full particles (FPs) under denaturing conditions. As shown in **Figure 4A**, mAb 4D6 specifically detected a single protein band corresponding to VP1, confirming that it recognizes a linear epitope located within VP1.

The specificity of mAb 4D6 was further evaluated by indirect ELISA using inactivated viral antigens from multiple enterovirus serotypes, including CV-A2, CV-A4, CV-A5, CV-A6, CV-A10, CV-A16, EV-A71, echovirus 11, and poliovirus. As shown in **Figure 4B**, mAb 4D6 reacted exclusively with CV-A5 antigen and exhibited no detectable cross-reactivity with other enteroviruses, demonstrating strict serotype specificity.

The binding activity of mAb 4D6 to CV-A5 was quantified by indirect ELISA. Nonlinear regression analysis revealed a half-maximal effective concentration (EC_50_) of 47.72 ng/mL (**Figure 4C**), indicating strong binding affinity of mAb 4D6 for CV-A5 particles.

The neutralizing activity of mAb 4D6 against CV-A5 strains representing different genotypes was further evaluated in RD cells. As summarized in **Table 2**, mAb 4D6 efficiently neutralized all tested CV-A5 strains, with comparable neutralizing concentrations differing by less than two-fold among clinical isolates, and exhibited slightly reduced activity against the fluorescent reporter virus CV-A5-r611-iLOV.

**Table 2:** Neutralizing activity of monoclonal antibody 4D6 against CV-A5 strains of different genotypes.

Virus strain	Neutralizing concentration (μg/mL)*	Virus input (CCID_50_/50 μL)
CV-A5-3474	1.23	100
CV-A5-3487	0.77	100
CV-A5-3490	0.89	100
CV-A5-611	0.93	100
CV-A5-r611-iLOV	1.45	100

*Neutralizing concentration represents the antibody concentration required to completely prevent virus-induced infection of RD cells, as determined by a fluorescence-based neutralization assay. Neutralization assays were performed using the indicated virus inputs. The fluorescent reporter virus CV-A5-r611-iLOV contains an iLOV insertion within the viral genome.

Collectively, these results demonstrate that mAb 4D6 is a CV-A5-specific neutralizing antibody targeting a linear epitope within VP1, with robust binding and neutralizing activity across multiple CV-A5 strains. The variable regions of the heavy and light chains of mAb 4D6 were successfully amplified and sequenced (data not shown), enabling subsequent epitope mapping and functional analyzes.

### 3.5 Identification of a novel linear neutralizing epitope recognized by mAb 4D6

**Figure 5:**
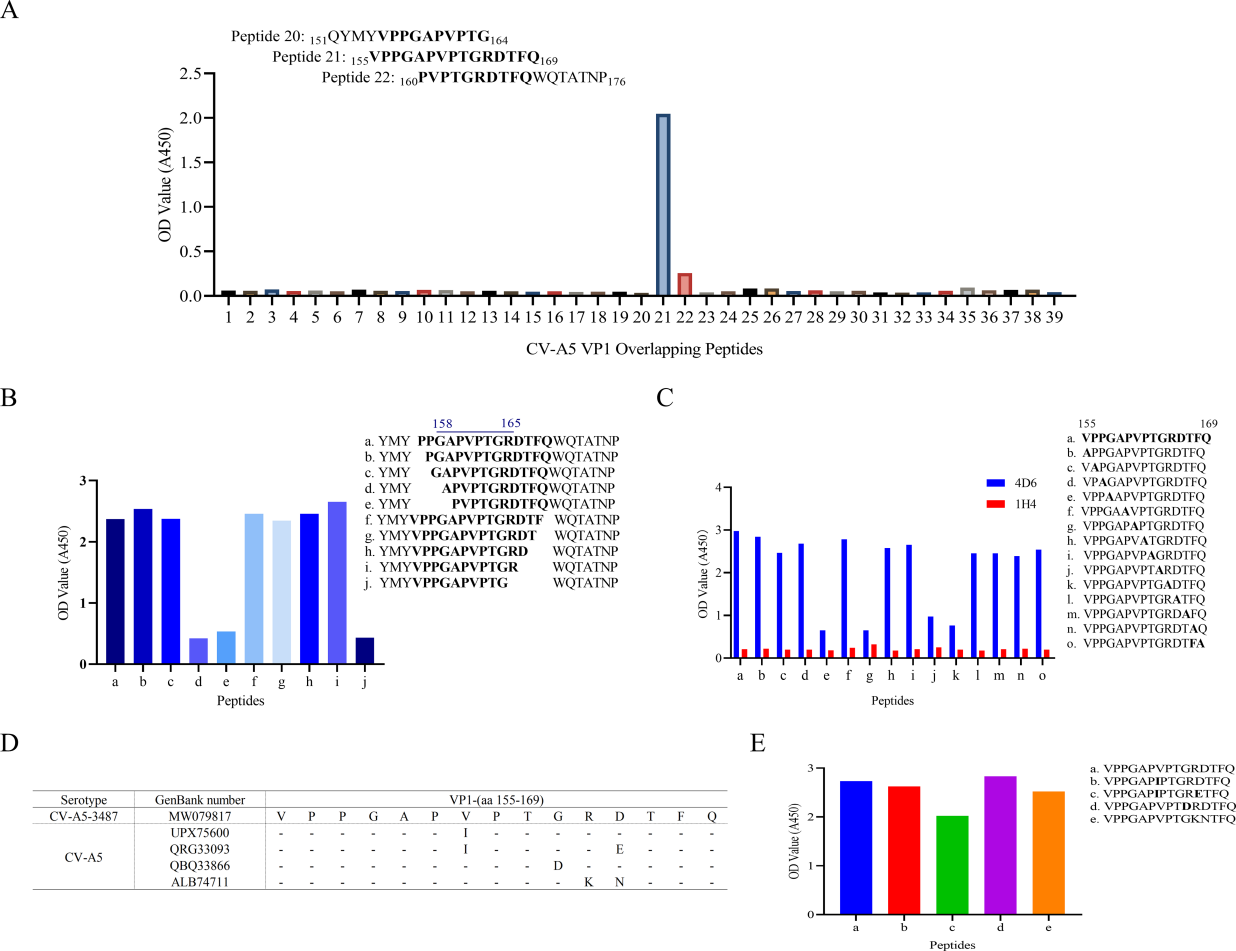
Epitope mapping of mAb 4D6 on CV-A5 VP1. **(A)** Peptide ELISA to map the binding site of mAb 4D6 on CV-A5 VP1. A set of 39 overlapping peptides covering the entire VP1 region (10 amino acids overlap) were tested for reactivity with mAb 4D6. Overlapping residues from peptides 20 to 22 are shown in bold. **(B)** Truncated peptides from the epitope region were tested in ELISA for binding reactivity to mAb 4D6. The inferred minimal epitope, from G158 to R165, is underlined. **(C)** Alanine scanning peptides spanning the epitope region were synthesized and tested in ELISA for reactivity with mAb 4D6. Mutated residues are indicated in bold. A control mAb (1H4) that recognizes a conserved, linear epitope on VP1 of enterovirus A species without neutralizing activity was included. **(D)** Sequence alignment of the identified epitope region (G158-R165) across different CV-A5 serotypes. Variant residues are indicated. **(E)** ELISA analysis to assess the binding activity of mAb 4D6 to peptides containing mutations from panel **(D)** Mutated residues are indicated in bold.

To precisely map the linear epitope recognized by mAb 4D6, a panel of 39 overlapping synthetic peptides spanning the entire VP1 protein of CV-A5 was initially screened by indirect ELISA. Each peptide was 14–18 amino acids in length, with adjacent peptides overlapping by 10 amino acids. This primary screening identified a single reactive region corresponding to VP1 residues 155-169 (**Figure 5A**, peptide 21).

To further delineate the minimal epitope required for antibody binding, a series of truncated peptides derived from this region were synthesized and analyzed by ELISA. As shown in **Figure 5B**, progressive truncation from either terminus revealed that the antibody-binding activity was retained within an 8-amino-acid core sequence spanning residues 158-165 (GAPVPTGR), which was defined as the minimal essential epitope recognized by mAb 4D6.

To identify critical residues involved in epitope recognition, alanine-scanning mutagenesis was performed across the minimal epitope. Substitution of Gly158, Val161, Gly164, or Arg165 with alanine resulted in a marked reduction or complete loss of mAb 4D6 binding, whereas substitutions at other positions had minimal effects (**Figure 5C**). These results indicate that these four residues play key roles in maintaining the structural integrity required for antibody recognition.

To assess the conservation of the identified epitope among circulating CV-A5 strains, sequence alignment of VP1 proteins retrieved from the NCBI enterovirus database was performed. The core epitope GAPVPTGR was found to be highly conserved, with an overall conservation rate of 90.5% among analyzed CV-A5 sequences (data not shown). Several naturally occurring variant motifs were identified, including V161I, V161I/D166E, G164D, and R165K/D166N (**Figure 5D**).

To determine whether these naturally occurring mutations affected antibody recognition, corresponding mutant peptides were synthesized and evaluated by ELISA. As shown in **Figure 5E**, mAb 4D6 retained substantial binding activity to peptides containing the V161I or G164D substitutions, as well as to peptides harboring the double mutations V161I/D166E and R165K/D166N. These results suggest that mAb 4D6 tolerates certain naturally occurring sequence variations within the epitope, supporting its broad reactivity among CV-A5 strains.

Collectively, these data identify VP1 residues 158-165 (GAPVPTGR) as a previously unreported, conserved linear neutralizing epitope of CV-A5 and establish mAb 4D6 as a linear epitope-specific antibody with potent neutralizing activity.

### 3.6 Protective efficacy of mAb 4D6 in a lethal CV-A5 mouse model

**Figure 6:**
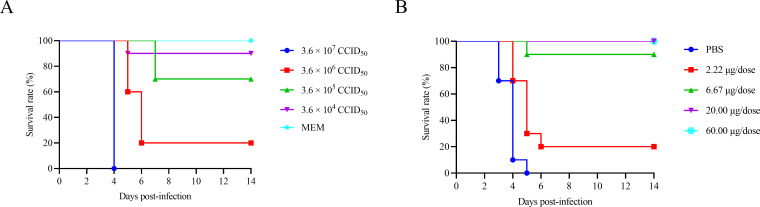
Protective efficacy of mAb 4D6 against lethal CV-A5 challenge. **(A)** Determination of LD_50_ for CV-A5–611 in 14-day-old Kunming mice. Mice were inoculated intraperitoneally (i.p.) with serial virus doses of CV-A5-611 (3.16 × 10^7^ to 3.16 × 10^4^ CCID_50_ per mouse), and the control group received MEM medium. **(B)** Passive protection of mice by mAb 4D6 following CV-A5–611 lethal challenge. Mice were i.p. inoculated with 3.16 × 10^7^ CCID_50_ of CV-A5-611 (44 LD_50_). Two hours later, mice were treated with 4D6 at doses ranging from 2.22 μg to 60 μg per mouse. The control group received CV-A5–611 challenge followed by PBS injection.

To assess the *in vivo* protective efficacy of mAb 4D6, the median lethal dose (LD_50_) of the mouse-adapted CV-A5–611 strain was first determined in 14-day-old Kunming mice. Groups of mice were challenged intraperitoneally with serial 10-fold dilutions of CV-A5–611 and monitored for survival for 14 days. Based on mortality data, the LD_50_ of CV-A5–611 was calculated to be 7.18 × 10^5^ CCID_50_ per mouse (**Figure 6A**).

For passive immunization experiments, 14-day-old Kunming mice were challenged intraperitoneally with CV-A5–611 at a dose of 3.16 × 10^7^ CCID_50_ per mouse, corresponding to 44 × LD_50_. Two hours after viral challenge, mice were administered mAb 4D6 intraperitoneally at doses of 60, 20, 6.67, or 2.22 μg per mouse, while control mice received phosphate-buffered saline (PBS) alone.

As shown in **Figure 6B**, mice in the PBS control group began to exhibit severe clinical symptoms, including hindlimb paralysis, at 4 days post-infection (dpi), and all mice succumbed to infection by 5 dpi. In contrast, administration of mAb 4D6 conferred dose-dependent protection against lethal CV-A5 challenge. Complete protection without observable clinical symptoms was achieved in mice receiving 60 μg or 20 μg of mAb 4D6. Partial protection was observed in the 6.67 μg group, whereas mice treated with 2.22 μg of mAb 4D6 exhibited minimal protection (**Figure 6B**).

Survival curve analysis revealed an improvement in survival for mice treated with mAb 4D6 at doses of ≥ 6.67 μg compared with the PBS control group (**Figure 6B**). These results demonstrate that mAb 4D6 provides effective post-exposure protection against lethal CV-A5 infection *in vivo*.

## 4 Discussion

Enteroviruses undergo multiple structural transitions during their life cycle, generating distinct particle forms including empty particles (EPs), mature full particles (FPs), uncoating intermediates (A-particles), and post-uncoating B-particles. It is well known that APs are difficult to harvest from the enterovirus-infected cells (23). Although these structural states have been described for several enteroviruses, the particle heterogeneity and its immunological relevance in Coxsackievirus A5 (CV-A5) has remained limited. In the present study, we successfully isolated EPs, FPs, and APs of CV-A5 and further identified two distinct populations of EPs (EP-1 and EP-2) with different buoyant densities, indicating previously unrecognized structural heterogeneity among CV-A5 empty particles. The biochemical and morphological properties of these particles were consistent with those reported for other picornaviruses, supporting the general conservation of particle maturation pathways across enterovirus species. Importantly, comparison of immunogenicity among the five particle types revealed that FPs elicited the strongest neutralizing antibody responses, whereas EP-1 exhibited the weakest immunogenicity. These results are consistent with previous studies showing that intact mature virions required for correct epitope presentation. Notably, EP-2 induced higher neutralizing responses than EP-1, despite both lacking genomic RNA, suggesting that subtle structural differences among empty particles can influence epitope presentation and immune recognition. In addition, we observed that EP-2 induced neutralizing antibody titers comparable to those of FP. We speculate that EP-2 may represent a distinct empty particle conformation with increased surface exposure of neutralizing epitopes, which may account for its enhanced immunogenicity. Artificially generated B-particles retained moderate immunogenicity, indicating that genome release and capsid expansion do not completely abolish antigenic determinants. Together, these results highlight particle heterogeneity as a critical determinant of immunogenicity and underscore the importance of maintaining native FP structure during vaccine antigen preparation. For CV-A5 vaccine development, minimizing non-native or structurally altered particles may be essential for achieving optimal neutralizing antibody responses.

Efficient generation of neutralizing monoclonal antibodies against enteroviruses is often limited by the predominance of non-neutralizing antibody responses elicited by conventional immunization strategies. To enhance the induction of functional antibodies, we employed an optimized immunization approach combining live virus priming with purified FP boosting, thereby mimicking natural infection while preserving native antigenic structures. This strategy has been shown to enhance the breadth and potency of antibody responses (24, 25). The proportion of neutralizing hybridomas (13.61%) was markedly increased, substantially exceeding the rates reported in previous study using purified particles alone (22). In addition, intraperitoneal immunization produced significantly higher binding and neutralizing antibody titer than oral administration, indicating that the route of immunization strongly influences the magnitude of humoral immune responses. The use of a fluorescent reporter virus further enabled rapid functional screening of neutralizing antibodies within 24–36 h, greatly accelerating antibody discovery compared with traditional cytopathic effect-based assays. This strategy not only improved screening efficiency but also reduced the likelihood of selecting non-neutralizing binding antibodies. Collectively, these methodological advances provide a practical framework for the rapid generation and identification of functional neutralizing antibodies against enteroviruses.

Consistent with the known antigenic architecture of enteroviruses, the majority of neutralizing antibodies obtained in this study recognized conformational epitopes. However, among the ten neutralizing monoclonal antibodies characterized, one antibody, mAb 4D6, was identified as a linear epitope-specific neutralizing antibody targeting VP1.

VP1 is a major surface-exposed capsid protein involved in receptor binding and uncoating and is a primary target of neutralizing antibodies across enterovirus species (26, 27). Structural and immunological studies of enterovirus A species members have shown that VP1 contains several surface-exposed loop regions, including the B-C loop (residues 97-105), E-F loop (residues 163-177), G-H loop (residues 208-225) (28). Many previously identified linear neutralizing epitopes of EV-A71, CV-A16, and CV-A10 are located within these flexible loop regions (12, 14, 15). These loops, often hydrophilic and elastic, are mutation-prone regions and contain the most important linear neutralizing epitopes with high specificity (29).

In this study, epitope mapping revealed that mAb 4D6 recognizes a linear epitope spanning VP1 residues 158-165 (GAPVPTGR), located within the broader 155–169 region. This epitope has not been previously reported in CV-A5. Alanine-scanning analysis identified key residues required for antibody binding, indicating the presence of a structurally constrained motif essential for epitope recognition. Sequence alignment demonstrated that this epitope is highly conserved (90.5%) among circulating CV-A5 strains, suggesting evolutionary constraint and supporting its importance as a neutralization determinant.

The identification of a conserved linear neutralizing epitope is of significance for vaccine and antibody design. Compared with conformational epitopes, linear epitopes are more amenable to peptide-based vaccine development, epitope-focused immunogen design, and antibody engineering. Importantly, mAb 4D6 retained binding activity to peptides containing naturally occurring sequence variations within this region, supporting its broad reactivity among CV-A5 strains. Similar conserved linear neutralizing epitopes have been reported in other enteroviruses and are often associated with potent cross-protective immune responses, highlighting their potential value as targets for rational vaccine design (30, 31).

*In vivo* passive transfer experiments further demonstrated that mAb 4D6 conferred dose-dependent protection against lethal CV-A5 infection, with complete protection achieved at higher doses even when administered after viral challenge. These findings indicate that targeting the identified VP1 linear epitope is sufficient to mediate effective neutralization and protection *in vivo*. To our knowledge, this is the first report describing a linear neutralizing epitope of CV-A5 that confers post-exposure protection in an animal model. While the precise mechanism of neutralization by mAb 4D6, such as inhibition of receptor binding, capsid destabilization, or interference with uncoating, remains to be elucidated, its robust protective efficacy highlights its potential as a candidate therapeutic antibody and supports the relevance of this epitope as a target for antibody-based intervention.

Several limitations of this study should be noted. First, the absence of a high-resolution CV-A5 capsid structure prevents direct visualization of the 4D6 epitope on the native virion, and its surface exposure is inferred based on structural homology with other Enterovirus A members (15, 32). Second, the molecular mechanism underlying neutralization by mAb 4D6 was not investigated and will require further structural and functional studies. Finally, although sequence conservation and functional assays suggest broad reactivity within CV-A5, cross-neutralization against other enterovirus A serotypes was not observed and remains an area for future exploration. Further structural analysis and epitope-focused vaccine studies will be necessary to determine whether broader protection can be achieved.

## 5 Conclusion

In summary, this study provides a systematic characterization of particle heterogeneity, immunogenicity, and antigenicity determinants of CV-A5. We demonstrate that mature full particles are the most effective in inducing neutralizing antibody responses and identify, for the first time, a conserved linear neutralizing epitope on VP1 that mediates potent *in vivo* protection. The strong protective efficacy of mAb 4D6 highlights the importance of this epitope as a target for therapeutic antibody development and rational vaccine design. These findings advance our understanding of CV-A5 immune recognition and offer valuable guidance for the development of vaccines and antibody-based therapeutics against emerging HFMD-associated enteroviruses.

## Data Availability

The original contributions presented in the study are included in the article/supplementary material. Further inquiries can be directed to the corresponding authors.
